# The IDH1 Mutation-Induced Oncometabolite, 2-Hydroxyglutarate, May Affect DNA Methylation and Expression of PD-L1 in Gliomas

**DOI:** 10.3389/fnmol.2018.00082

**Published:** 2018-03-28

**Authors:** Luyan Mu, Yu Long, Changlin Yang, Linchun Jin, Haipeng Tao, Haitao Ge, Yifan E. Chang, Aida Karachi, Paul S. Kubilis, Gabriel De Leon, Jiping Qi, Elias J. Sayour, Duane A. Mitchell, Zhiguo Lin, Jianping Huang

**Affiliations:** ^1^The Fourth Section of Department of Neurosurgery, The First Affiliated Hospital, Harbin Medical University, Harbin, China; ^2^The First Section of Department of Neurosurgery, The Fourth Affiliated Hospital, Harbin Medical University, Harbin, China; ^3^Lillian S. Wells Department of Neurosurgery, University of Florida, Gainesville, FL, United States; ^4^Department of Pathology, The First Affiliated Hospital, Harbin Medical University, Harbin, China

**Keywords:** gliomas, immune checkpoint, PD-L1, DNA methylation, IDH mutation, 2-hydroxyglutarate

## Abstract

**Background:** Malignant gliomas are heterogeneous brain tumors with the potential for aggressive disease progression, as influenced by suppressive immunoediting. Given the success and enhanced potential of immune-checkpoint inhibitors in immunotherapy, we focused on the connections between genetic alterations affected by IDH1 mutations and immunological landscape changes and PDL-1 expression in gliomas.

**Methods:** Paired surgically resected tumors from lower-grade gliomas (LGGs) and glioblastomas (GBM) were investigated, and a genetic analysis of patients' primary tumor samples culled from TCGA datasets was performed.

**Results:** The results demonstrate that when compared with IDH1-mutant tumors, IDH1 wildtype tumors represent an immunosuppression landscape and elevated levels of PD-L1 expression. DNA hypo-methylation of the PD-L1 gene, as well as high gene and protein expressions, were observed in the wildtype tumors. We also found that quantitative levels of IDH1 mutant proteins were positively associated with recurrence-free survival (RFS). A key product of the IDH1 mutation (2-hydroxyglutarate) was found to transiently increase DNA methylation and suppress PD-L1 expression.

**Conclusions:** IDH1 mutations impact the immune landscape of gliomas by affecting immune infiltrations and manipulating checkpoint ligand PD-L1 expression. Applications of immune checkpoint inhibitors may be beneficial for chemoradiation-insensitive IDH1-wildtype gliomas.

## Introduction

PD-1 and its ligands, programmed death-ligand 1 (PD-L1) (Dong et al., 1999) and PD-L2 (Latchman et al., 2001), were shown to be part of an important family of molecules involved in tumor immunosuppression (Freeman et al., 2000; Brahmer et al., 2012). Studying how these molecules are positioned in the immunosuppressive landscape of gliomas, as based on the information provided by tumor subsets, may provide a better understanding of the application of immune-checkpoint inhibitors. For instance, patients with IDH1 wildtype LGGs suffer from the most aggressive form of the disease and have overall survival (OS) that are strikingly similar to those of patients with GBMs, with a median overall survival of ~1 year; treatment options are confined to surgery and chemo/radiation with dismal efficacy in these patients (Eckel-Passow et al., 2015; Suzuki et al., 2015; Ceccarelli et al., 2016). As a result, this population of patients is in desperate need of novel treatment approaches, such as the use of immune-checkpoint inhibitors or combinations featuring other treatment modalities.

Mounting evidence indicates that tumor-associated mutations represent key factors that affect immunogenicity in tumors. In gliomas, cytosolic NADP+-dependent isocitrate dehydrogenase 1 (IDH1) became a more valuable determinant of disease characteristics, sensitivity to chemo/radiation therapy, and OS in glioma patients when compared with histological grades (Ichimura et al., 2009; Verhaak et al., 2010; Rohle et al., 2013). Approximately 90% of the mutations occur in the IDH1 gene (Yan et al., 2009). There is evidence to support the notion that IDH1 mutations may be associated with glioma susceptibility (Amary et al., 2011; Pansuriya et al., 2011). Nearly all IDH1-mutant gliomas contain methylated O-6-methylguanine-DNA methyltransferase (*MGMT*) genes, which sensitize tumors to temozolomide (TMZ) therapy (Hegi et al., 2005; Mulholland et al., 2012).

Although IDH1 mutations that encode tumor-specific epitopes can be immunologically exploited, the tumor microenvironment, including regulatory T cells (Tregs), myeloid-derived suppressor cells (MDSCs), and tumor-associated macrophages (TAMs) (Almand et al., 2001; Dieckmann et al., 2001; Chen and Mellman, 2013; Zhou et al., 2015), may suppress antitumor immunity and play a critical role in cancer development and progression. Thus far, few reports have been published on the association between IDH gene mutations and tumor immune cell infiltration, such as CD8+ T cells (Kohanbash et al., 2017), macrophages, and CD4+ T, B, and dendritic cells (Amankulor et al., 2017), as well as the connections between mutations and immune-checkpoint molecules (Berghoff et al., 2017; Hodges et al., 2017).

In this study, we provide more evidence to support the finding that the regulation of PD-L1 also known as cluster of differentiation 274 (CD274) glioma expression is directly associated with tumor IDH1 mutation status. The global immune landscapes of the IDH1 wildtype and mutant tumors were found to be significantly different. These data support the rationale that stratifying gliomas by IDH1 mutation status enables more precisely targeted immunotherapeutics, such as checkpoint inhibitors and improved outcomes for patients with chemo/radiotherapy-resistant wildtype IDH1 gliomas.

## Materials and methods

### Patient population

Paired (primary and recurrent/secondary) surgically resected tumors from 35 adult patients who were diagnosed with primary diffuse astrocytoma (DA) or oligodendrocytoma, as well as 15 primary GBMs before and after recurrence, were collected in three affiliated hospitals of Harbin Medical University. These patients were admitted to the hospitals between 2005 and 2014. All specimens underwent a full pathological review. The patients were therapy-naïve before surgery on the primary tumor. After surgery, these patients were given either standard radiotherapy (60 Gy) or concomitant adjuvant chemotherapy [temozolomide (TMZ) or others] (Table S2). All subjects gave written informed consent in accordance with the Declaration of Helsinki, and the research protocols were reviewed and approved by the Committee on Human Research at Harbin Medical University, Harbin, China.

### Immunohistochemistry staining and quantification

The immunohistochemistry (IHC) sample slides were reviewed by three neuropathologists, and a systematic neuropathological review was based on the 2007 World Health Organization (WHO) classification of central nervous system tumors (Louis et al., 2007). The tissue regions with the highest degree of inflammation, such as necrosis and hemorrhage, were precluded. Formalin-fixed paraffin-embedded (FFPE) specimens were stained with antibodies against CD3 (clone LN10, dilution 1:200; Quanhui, China), CD4 (clone UMAB64, 1:50 dilution, ZSGB-BIO, China), CD8 (clone SP16, 1:100, dilution; ZSGB-BIO), Foxp3 (clone mAbcam 450, 1:50 dilution; Abcam, Cambridge, MA, USA), CD68 (clone KP1, 1:100 dilution; ZSGB-BIO), CD163 (clone 10D6, 1:200 dilution; Quanhui), and IDH1 R132H (clone H09, 1:50 dilution; Maxim, China). Each staining batch included a negative control without the primary antibody and/or a biological negative control of normal brain tissue obtained from donors who died of a stroke, and/or a positive control (tonsil specimens). The samples were then incubated with the horseradish peroxidase-labeled secondary antibody in the IHC kit (KIT-5930, Maxim, China). Diaminobenzidine was used for color development, and hematoxylin was used as a counterstain. IHC analyses were then performed with a quantitative approach under a light microscope (Leica; Leica Optical Co. Ltd. Wetzlar, Germany). The immune cell (T cell subset markers CD4, CD8, Foxp3+ cells, and macrophage markers CD68 and CD163) infiltrates in the tumors were counted in three large regions. In each region, a mean of 10 consecutive high-power fields was recorded by experienced pathologists who were blinded to the patients' clinical information. The average counts of the three regions were used for the final report. The average scores counted by the pathologists were recorded as the final result. When there was a large difference in scores between observers, the score was re-evaluated to reach an agreement (Mu et al., 2017). To quantify the protein levels of mutant IDH1, as well as the PD-L1 expression, all captured 20X magnification fields of IHC were imported to Image-Pro Premier 9.1 to measure each image's integrated optical density (IOD). The DAB plugin was used to determine the IDO and area. Blank and vascular areas in the images were excluded. Each IOD was then normalized to actual areas. Each patient's results were used in the statistical analysis.

### TCGA data analysis

Data from The Cancer Genome Atlas (TCGA) used in this study were downloaded from https://cancergenome.nih.gov/. Gene expressions by RNA-seq (IlluminaHiSeq version 2) were used. All patients with available vital status information were subgrouped by median gene expression; then, Kaplan–Meier survival analysis was used. The differential gene expression was also observed by subgrouping patients according to their IDH mutation and chromosome 1p/19q status (Cancer Genome Atlas Research Network et al., 2015). The β-values of DNA methylation were derived from the UCSC Cancer Browser and were already offset by −0.5 to shift the whole dataset to values between −0.5 to +0.5, as described on the UCSC Cancer Browser website. The probes we chose for promoter regions of PD-L1 were determined by the Ensembl Genome Browser.

### Detection of IDH1 mutation by gene sequencing

DNA sequencing was performed to validate the accuracy of IDH1 mutation detection using an anti-IDH1 monoclonal antibody. Genomic DNA was isolated from FFPE tumor tissue using a QIAamp DNA Mini Kit (Qiagen Inc., Carlsbad, CA, USA). Introns and intron/exon boundaries of exon 4 of the IDH1 gene were amplified by polymerase chain reaction (PCR) using JumpStart RED Taq Ready Mix (Sigma-Aldrich Co., St. Louis, MO, USA) according to the manufacturer's instructions. PCR products were sequenced on a 3130 Genetic Analyzer (Applied Biosystems, Foster City, CA, USA) following standard procedures. The primers used for PCR amplification and sequencing were IDH1 exon 4 F-TATTGCCTCTATCTGGTGAAA and IDH1 exon 4 R-AATGGGTGTAGATACCAAA AG, 706 bps. The reaction was conducted for 40 cycles. The sequencing result was evaluated using Chromas software (Technelysium Pty Ltd.).

### DNA methylation

Genomic DNA was isolated from the tumor lines using a DNA isolation kit according to the manufacturer's instructions (Roche Applied Science, Berlin, Germany). PD-L1 methylation status was determined by the methylation-specific (Ms) PCR and pyrosequencing, using the following primers for sequence amplification: for cg15837913: sense, 5′-GGTAGAATATTAGGGATTTTGAGTATTT-3′; anti-sense, 5′-CAACAACAAA CCCATATAACTTTAAT-3′. For cg19724470: sense, 5′-TTGATGTTAGGTTGGAGGT TTG-3; anti-sense, 5′-AAACTCCTCCATTCCTCTTT-3′. The analysis was performed by Beijing Genomics Institute (BGI) in China. The information related to the primers used in this study is displayed in Table S3.

### qPCR

The mRNA was reverse transcribed into cDNA with a reverse-transcription kit (Promega Biotech). Quantitative real-time PCR was performed with Universal Master Mix (Chembase, Moscow, Russia). cDNA (50 ng) was used to determine the relative amounts of PD-L1 mRNA by real-time PCR (MAX3000 Sequence-Detection System, Chembase) using the following primers: sense, 5′-TCTGGACAAGCAGTGACCATC-3′; anti-sense, 5′-GTGTTGATTCTC AGTGTGCTGG-3′. The reaction was conducted for 40 cycles.

### Statistical analyses

Bivariate comparisons of binary data, small sets of binary data, and continuous data were performed using *t*-tests or the Mann–Whitney *U-*test, respectively. Cox proportional hazards models were used to evaluate hazard ratios (HRs). Kaplan–Meier survival analysis was used to determine the survival of the two indicated groups, and the results were analyzed with the log-rank test. Significance was established as follows: ^*^P < 0.05, ^**^P < 0.01, and ^***^P < 0.001.

## Results

### Creation of an immunosuppressive genetic landscape in primary gliomas is linked with the IDH1 wildtype

**Figure 1:**
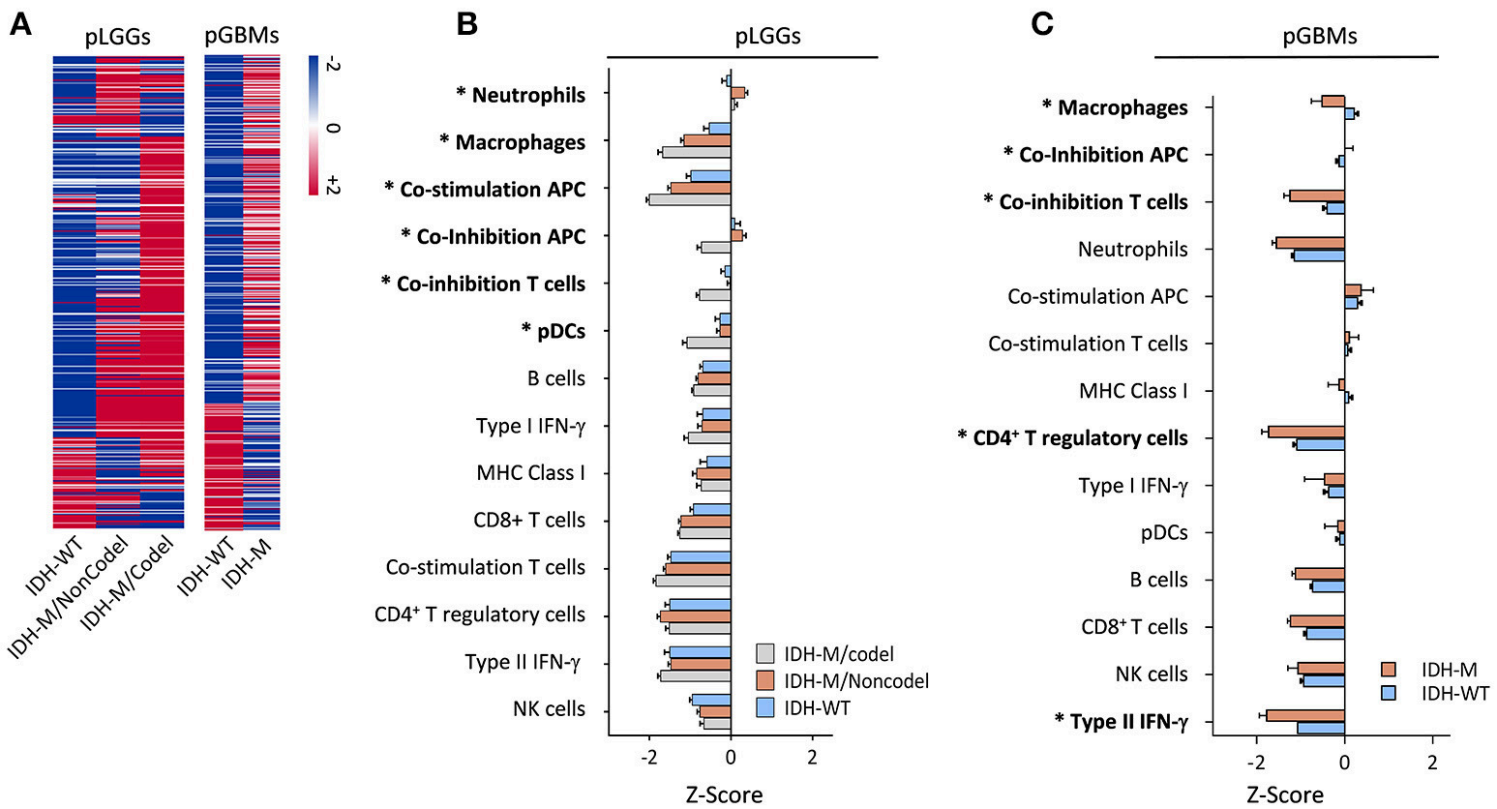
The immunological gene profile in primary LGG subgroups and GBMs with or without IDH1 mutations. **(A)** Hierarchical cluster of cancer immune-related genes among three LGG subgroups (left panel) and GBMs with or without an IDH1 mutation (right panel). RNA-Seq data from TCGA were analyzed and 750 cancer immune-associated genes (Cesano, 2015) were used to perform the hierarchical clustering according to the nCounter® PanCancer Immune Profiling Panel. These genes were imported to log2 normalization and centered by the mean gene expression value. **(B,C)** Differences in local immune infiltrates and cytolytic activity in the tumors was based on the terminology and data analysis used in a previous report (Rooney et al., 2015) for the three subgroups of LGGs and GBMs with or without the mutation. These tumor-related, local immune cell subtype/process-associated genes were clustered using Pearson's correlation among the subtypes. The enrichment scores (Z-scores) of each subgroup were plotted. The LGG patients were subdivided according to IDH1 mutation status and complete deletion of chromosome 1p/19q. Asterisks (^*^) indicate a significant difference found between the IDH1wildtype and -mutant tumors. We used mixed-effects analysis of variance (ANOVA) to screen across 14 cell types to assess differences in response among tumor types. The subject was modeled as a random effect nested within tumor type, and tumor type and cell type were both modeled as fixed effects. We specifically compared the IDH-WT tumor type mean to the average of the two IDH-mutated tumor type means within each cell type and assessed FDR-adjusted *P*-values at FDR = 1%.

Our original attempt was to identify the immune marker(s) that can help determine glioma progression using surgically resected tumors in newly diagnosed glioma patients. Paired samples were derived from patients with three categories of tumor recurrence; specifically, LGG recurred to LGG (LGG-LGG), LGG recurred to GBM (LGG-GBM), and GBM recurred to GBM (GBM-GBM) were evaluated (Mu et al., 2017). Three paired samples randomly selected from those samples were also analyzed for genetic profile changes upon tumor progression by comparing infiltrating immune cell populations in primary and recurrent tumors (Mu et al., 2017). Interestingly, significant alterations affected by tumor progression were found in the LGG to LGG and LGG to GBM recurrence, but not for the GBM to GBM recurrence, and the changes were primarily shown in the CD4+ T cell compartment (Table S1), suggesting that immune infiltrations link with glioma progression. Mounting evidence suggests that LGG progression is highly associated with IDH1 mutations and their related genetic abnormalities, such as TP53 mutation or total 1p/19q loss (Ichimura et al., 2009; Suzuki et al., 2015). We therefore sought to examine IDH1 mutation status (the predominant IDH1 mutation) in response to immune landscaping in primary gliomas using RNA-seq datasets culled from TCGA. A total of 750 cancer immune-associated genes were used to perform hierarchical clustering according to the nCounter® PanCancer Immune Profiling Panel https://www.nanostring.com/products/gene-expression-panels/hallmarks-cancer-gene-expression-panel-collection/pancancer-immune-profiling-panel (Cesano, 2015). The results showed that there were distinct distributions in gene expression between IDH1-mutant and wildtype tumors in both LGG and GBM (Figure 1A). Next, we characterized infiltrating immune subsets in these primary tumors and found that immune suppressive cell populations, such as macrophages, co-inhibition APC, co-inhibition T cells, and Tregs, dominate IDH1 wildtype tumors (Figures 1B,C). These results suggest that the IDH1 wildtype is associated with elevated immunosuppression when compared with the mutant tumors, especially when compared with the IDH1 mutation with 1p/19q codeletion in gliomas at the genetic level.

### More immunosuppressive immune cell infiltrates were found in IDH1 wildtype tumors

**Figure 2:**
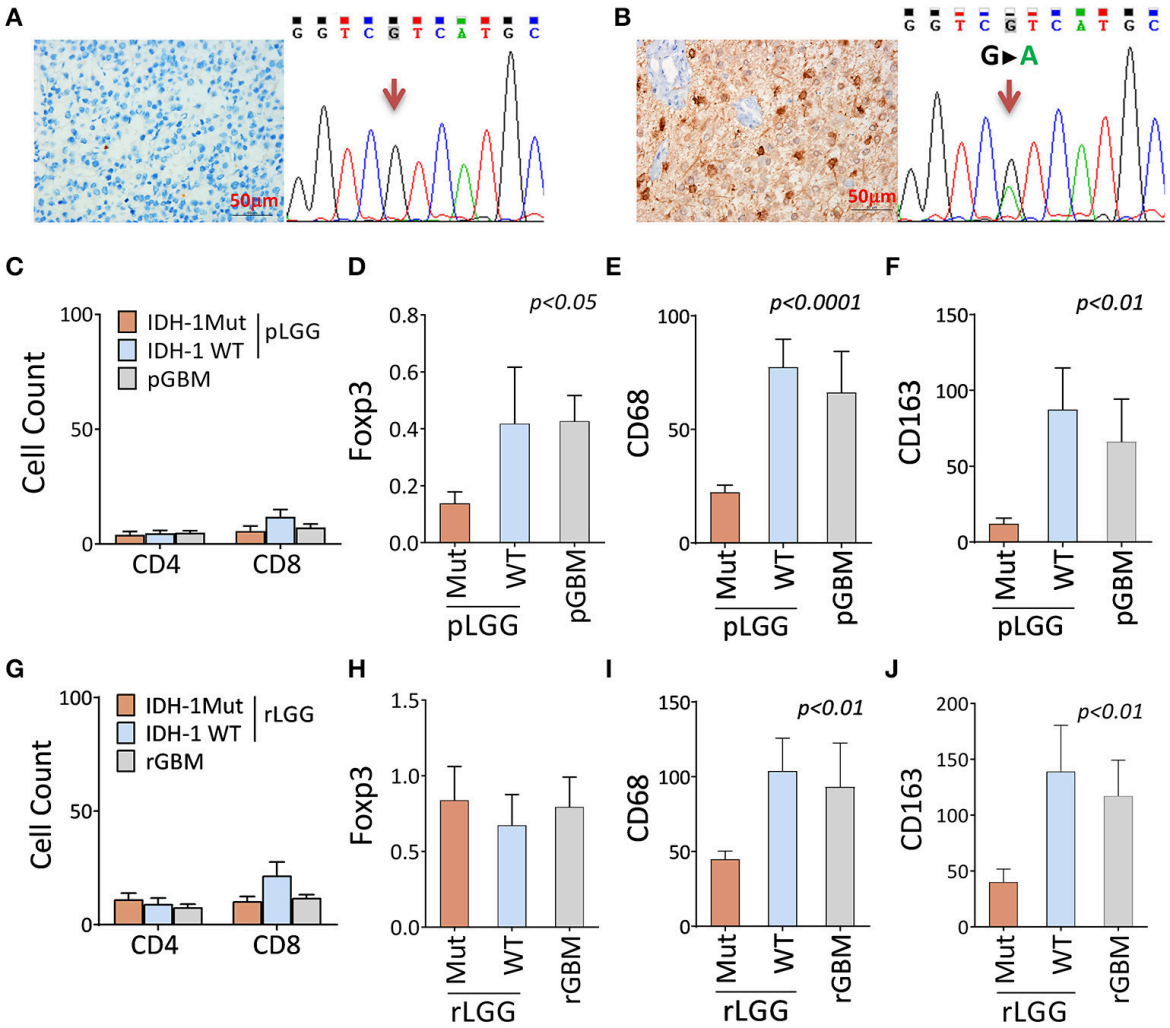
There were fewer immune-suppressive cell infiltrates in tumors with the IDH1 mutation compared with the IDH1 wildtype in primary and recurrent/secondary tumors. **(A,B)** Identification of the IDH1 mutation by DNA sequencing and antibody staining. A representative result of the IDH1 mutation, as determined by the anti-IDH1 mutation (R132H)-specific antibody and DNA sequence. **(C–F)** The immune infiltrates between patients with and without IDH1 mutation in primary LGG (pLGG) and GBM (pGBM) for CD4+, CD8+, and Foxp3+ T cells and CD68 and CD163 macrophages. **(G–J)** The immune infiltrates differ between patients with and without IDH1 mutations in recurrent LGG (rLGG) and GBM (rGBM) for the same markers. A total of 35 pairs of LGGs and 15 pairs of GBMs (pre- and post-tumor progression, as described in Table S2) were analyzed by immunohistochemistry staining for T cell subset makers (CD4, CD8, and Foxp3+ cells) and macrophage markers (CD68 and CD163). The immune infiltrates in the tumors were counted in three large regions. In each region, a mean of 10 consecutive high-power fields was recorded by experienced pathologists who were blinded to the patients' clinical information. The average counts of the three regions were used for the final report. The difference among the three groups was analyzed using one-way ANOVA. Any difference among group means and post hoc pairwise comparisons among tumor types were performed using FDR-adjusted *P*-values.

We next sought to test whether it was also true that a different immune landscape occurred at the cellular level. Markers defining immune subsets (CD3, CD4, CD8, Foxp3, CD68, and CD163) were stained for the patients' paired tumor samples, as described in Table S2 and Figures S1 A–F, and a comparison was performed based on IDH1 mutation status (only the IDH1-specific antibody was available) (Figures 2A,B). The results showed no significant changes in CD4+ and CD8+ T cell infiltration between IDH1-mutant and wildtype tumors (Figure 2C). The immunosuppression cell populations (Foxp3 [*P* < 0.05], CD68+ [*P* < 0.02], and CD163+ [*P* < 0.005] cells) were significantly lower in primary LGG (pLGGs) with the IDH1 mutation compared with the IDH1 wildtype tumors. The levels of these cells were found to be similar to those in primary GBMs (pGBMs) (Figures 2D–F). A similar trend (except for Foxp3) was also noted in recurrent tumors (Figures 2G–J). These results confirm that the IDH1 wildtype is associated with an immunosuppressive landscape at the cellular level.

### The levels of a mutant IDH1 protein associated with clinical outcomes in gliomas

**Figure 3:**
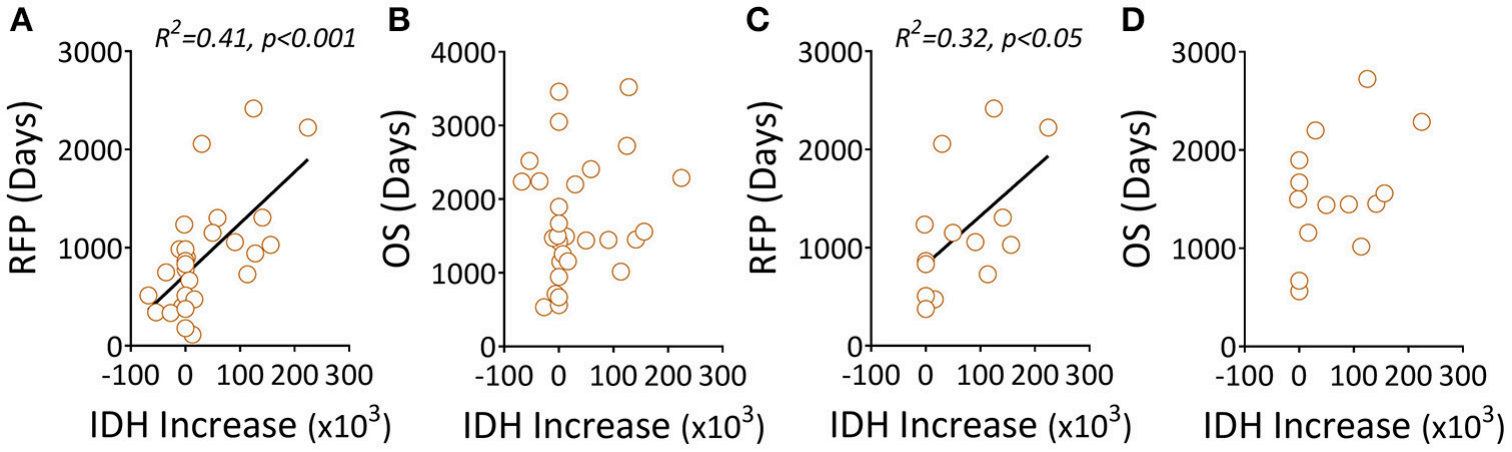
Elevated levels of a mutant IDH1 protein associated with clinical outcomes in gliomas. **(A,B)** Recurrent tumors, including LGG-LGG and LGG-GBM, were evaluated for correlations between mutant IDH1 protein changes (before and after recurrence) and RFS/OS. **(C,D)** The data only show recurrent LGG-GBM tumors. Quantitation of mutant IDH1 protein expression in tumor samples was performed using Image Plus Premier 9.1 software. The relative values of the mutant protein in each tumor are the mean density of ten 20× magnification fields. The IDH1 increase was the difference between recurrent and primary tumors featuring the IDH1 mutant protein. We used linear regression to estimate linear trend lines and R2 to test the strength of the association.

The mutation status of the IDH1 gene was shown to correlate with outcomes in patients with gliomas using mostly unpaired patient samples. We found a similar trend in this study using patients' paired samples; specifically, IDH1 mutation status was strongly correlated with patients' prolonged RFS (Figures S2A,B). Interestingly, we also found that quantitatively, the increased level of the mutant IDH1 protein (Figures S3 A,B) was positively associated with prolonged RFS, not OS (Figures 3A,B) in all three groups (Figures 3C,D).

### The genetic expression levels of PD-L1 is associated with poor survival in LGGs

**Figure 4:**
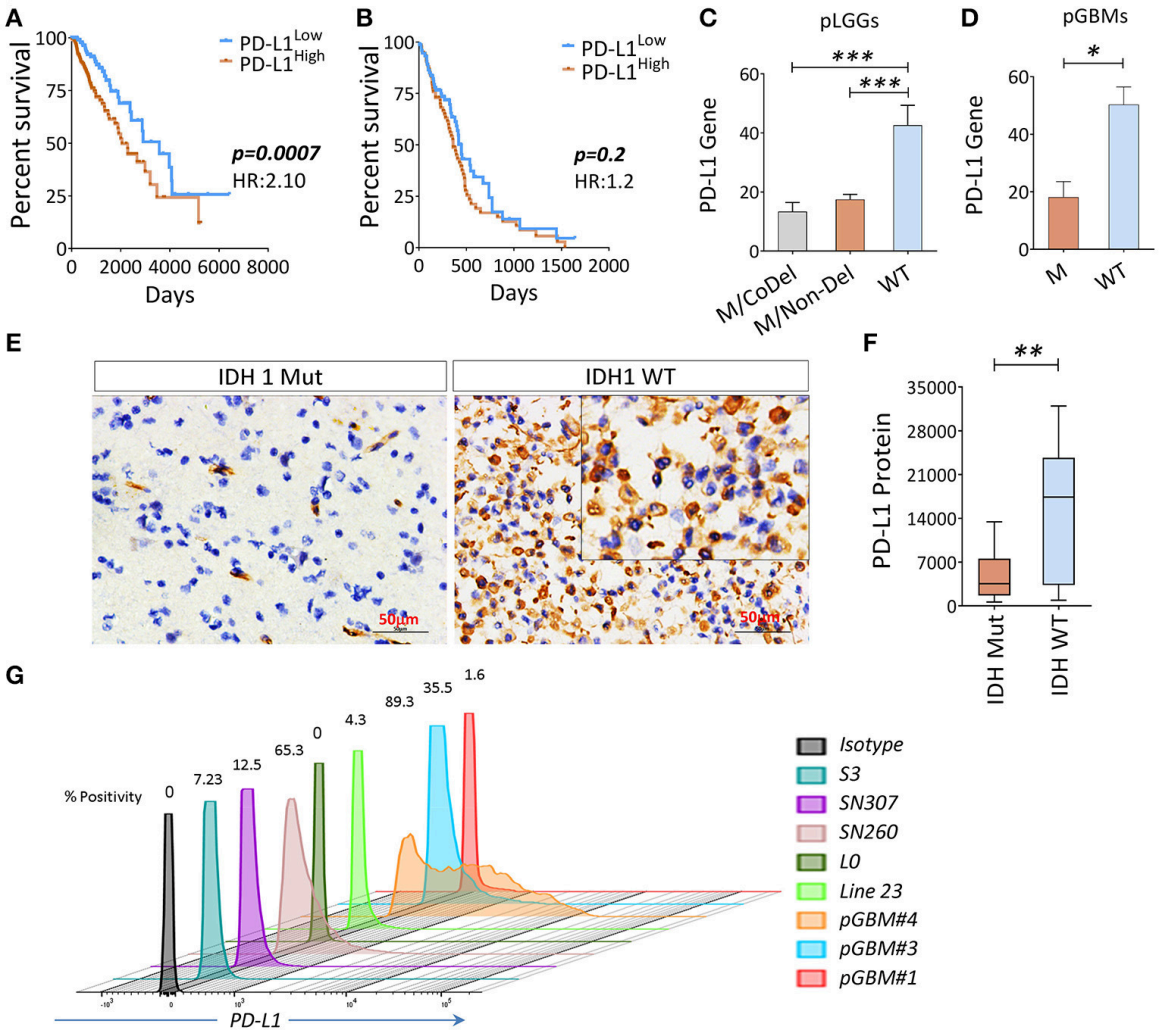
The association between PD-L1 gene expression (PD-L1) and OS in gliomas and wildtype tumors expressed a higher level of PD-L1. **(A)** The PD-L1 expression is inversely correlated with OS in pLGGs; **(B)** no similar trend was found in pGBMs. The RNAseq data of 282 primary LGGs and 151 primary GBMs with IDH mutation status and patients' OS culled from TCGA were analyzed. Median values were used as a cut-off point in each comparison. The Kaplan–Meier survival curves and the log-rank test were used to compare the groups in terms of OS and hazard ratios (HRs). **(C,D)** PD-L1 gene and PD-L1 expression among three LGG subgroups, as well as GBM with (WT) or without (M) IDH mutation. **(E,F)** IHC staining of PD-L1. Surgically resected tumors of mutant (*n* = 13) or wildtype (*n* = 14) LGGs were stained with a PD-L1 antibody; the quantitation of PD-L1 was analyzed using Image Plus Premier 9.1 software. **(G)** The PD-L1 expression on primary glioma lines; the PD-L1 expression on these cell lines was determined by FACS analysis and positivity was calculated using the isotype as a control. The difference between the two groups was analyzed using the Mann–Whitney *U*-test.

Since the PD-L1 gene was included on the list of genes along with co-inhibitory APC and/or co-inhibitory T cells in the analysis shown in Figures 1B,C, and the PD-L1 in tumor cells was sufficient for immune evasion in immunogenic tumors (Chen and Mellman, 2013). We therefore sought to evaluate this gene and patients' clinical outcomes. We found that PD-L1 was inversely associated with OS in pLGGs, but not in pGBMs (Figures 4A,B); however, IDH1 wildtype tumors expressed relatively higher levels of PD-L1 in both pLGGs and pGBMs when compared with the mutant tumors (Figures 4C,D). The same results were found at the protein level for PD-L1 in resected pLGGs (Figures 4E,F) compared with IDH1 wildtypes. In addition, some glioma cell lines, including primary GBM lines, were found to express PD-L1 (Figure 4G). There was no PD-L1 protein expression detected in CD163+ macrophages, a predominant immune population within tumors (Ge et al., 2017) (Figure S4). These data indicate that PD-L1 is primarily expressed in IDH1 wildtype tumors.

### DNA hyper-methylation of the PD-L1 gene affected by 2HG

**Figure 5:**
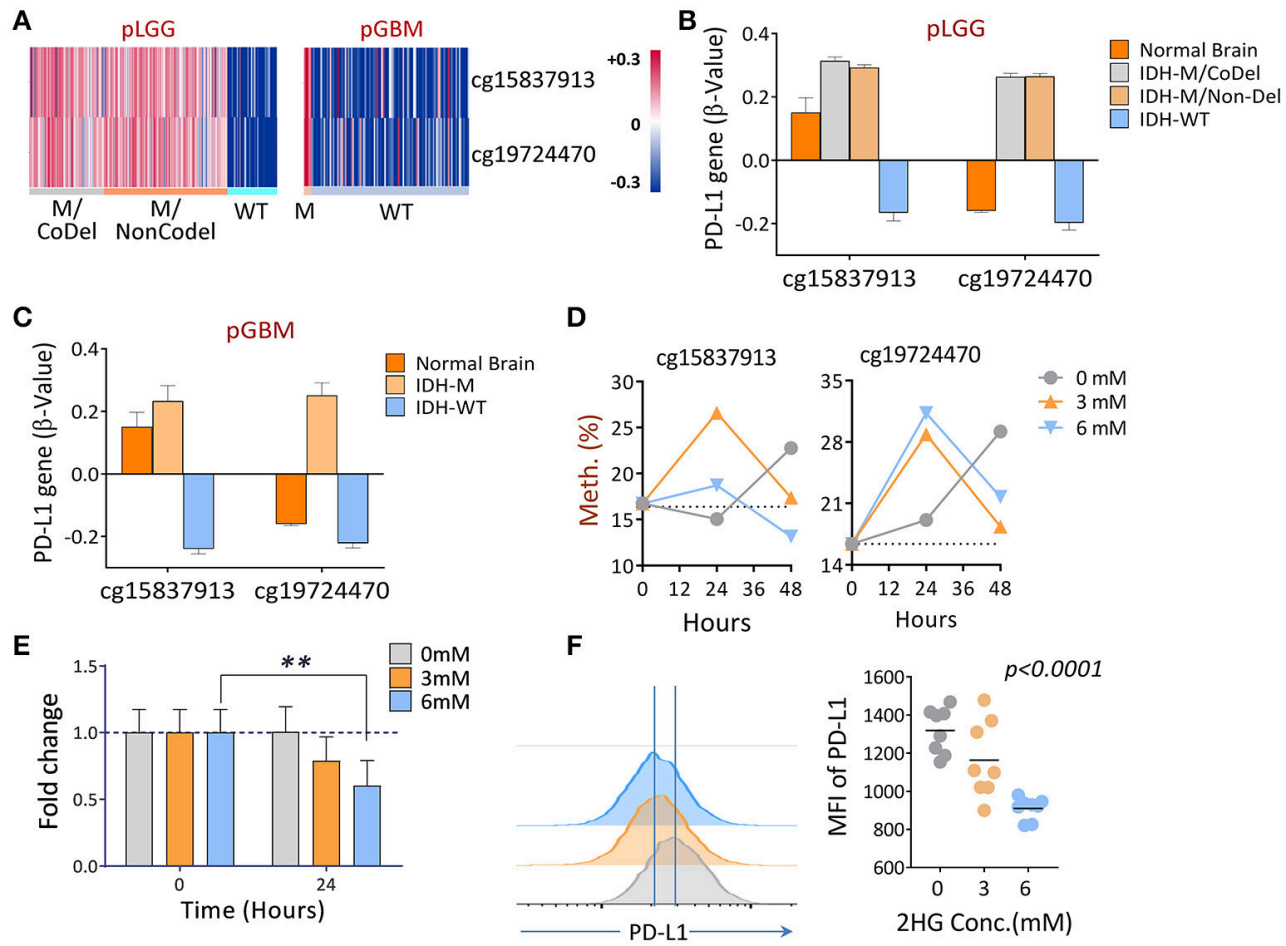
The differential DNA methylation of PD-L1 between IDH1 mutant and wildtype tumors in primary gliomas. **(A)** Hierarchical cluster of DNA methylation (cg15837913, cg19724470) measured by the β-values between IDH1 mutant and wildtype tumors is shown. **(B,C)** Quantitative β-values in different subgroups of primary gliomas. DNA methylation and IDH mutation/1p/19q codeletion information of primary LGG and GBM patients were derived from a TCGA Human Methylation 450k Array, as well as using the supplementary data from a previous publication (Suzuki et al., 2015). The β-values of each methylation probe were already offset by −0.5. **(D)** Adding 2-HG *in vitro* increased the DNA methylations of PD-L1. U87, an IDH wildtype, and a PD-L1-positive GBM line was cultured in medium containing indicated concentrations of 2HG, and the DNA was isolated at 24 and 48 h, respectively; the levels of methylation at baseline and 24 h were determined by pyrosequencing. **(E,F)** Adding 2HG transiently reduced gene (24 h) and protein (48 h) expression of PD-L1. The same cells described in **(D)** were also prepared for mRNA-qPCR and FACS analysis, and the experiments were repeated three times. The difference was determined using paired *t*-tests.

Since tumor-associated DNA methylation is a key epigenetic alteration that manipulates gene expression patterns which, in turn, can aid in tumor progression (Das and Singal, 2004; Ehrlich, 2009), we evaluated the DNA methylation profile of PD-L1 in primary LGGs and GBMs using normal brain tissues as controls to determine if there are glioma-specific DNA hyper- or hypo-methylation resulting in gene expression modulation. The promoter methylation profiles of the PD-L1 gene among the three subgroups of LGGs, as well as GBMs between the IDH1-mutant and wildtype tumors and healthy individuals were analyzed using the following TCGA datasets: LGG DNA methylation (Methylation450k) and GBM DNA methylation (Methylation450k). In the datasets, 5 probes within CpG islands were used to detect DNA methylations for PD-L1. Using normal brain tissues as controls, two of the probes within the PD-L1 promoter, cg15837913 and cg19724470, were found to be significantly different between normal brains and tumors, and the status of methylation also differed between IDH1-mutant and wildtype tumors (Figures 5A–C). Thereafter, the two fragments were of prime focus in the following experiments. Since mutations in the IDH1 gene results in the production of 2HG (Dang et al., 2009), we tested the DNA methylation status of PD-L1 of an IDH1 wildtype GBM line U87 when 2HG (0, 3, or 6 mM) was added daily to the cell culture, based on previously published report (Bralten et al., 2011). We found that the DNA methylation of PD-L1 was transiently increased in both CpG sites 24 h after adding 2HG, but it unexpectedly reversed in 48 h, and also, the non-treated cells showed a surge of beta-value at 48 h (Figure 5D). No definitive explanation for why these occurred in longer cultures. Since alterations in DNA methylation are dynamic, the mechanism of how they dictate spatial and temporal gene expression programs is still unclear (Smith and Meissner, 2013), we can only speculate that 2HG inhibits the cell proliferation (Bralten et al., 2011), which potentially affects the gene methylation status. In addition, changing culture condition by increased cell density may also be an influential factor. Nevertheless, significantly decreased gene and protein expression was observed (Figures 5E,F). These data imply that IDH1 mutations are directly involved in regulating PD-L1 expression in gliomas.

## Discussion

Since genetic alterations in tumors constitute a major source of tumor-specific antigens that can mediate immunological recognition, cancer immunotherapy can be developed to provide unique and precise tumor-specific therapy (Rosenberg et al., 2008; Restifo et al., 2012). However, tumors almost always resist immune attacks by creating a dominant immunosuppressive environment (Almand et al., 2001; Rabinovich et al., 2007; Postow et al., 2015). Targeting immunosuppressive pathways leads to patient response, even if patients have advanced disease and or have been heavily treated previously (Brahmer et al., 2012; Ghirelli and Hagemann, 2013). Thus, understanding the glioma immune microenvironment is indispensable for designing effective immunotherapeutic strategies.

The original aim of this study is to identify immune cell populations or immune markers in primary tumors that can be used to predict future disease progression in LGG patients. Coincidentally, we found that primary tumors with IDH1 mutations generally have lower levels of immune infiltrates compared with IDH1 wildtype tumors, including GBMs, which showed significantly greater numbers of immunosuppressive tumor-infiltrating cells, such as Tregs and TAMs. These results imply that IDH1-mutant tumors may lack particular key regulators for the recruitment, retention, promotion, and differentiation of immunosuppressive infiltrates. This prediction was later partly confirmed by the most recent reports that IDH mutations suppress STAT1 and CD8+ T cell accumulation and downregulate leukocyte chemotaxis in gliomas (Amankulor et al., 2017; Kohanbash et al., 2017). Additionally, no difference was found in the overall tumor mutation load between IDH1 wildtype and mutant tumors (Hodges et al., 2017), suggesting that more underlying mechanisms are involved in generating the differential immune landscape.

The analysis of a large cohort of surgically resected primary LGGs and GBMs culled from the TCGA dataset indicates that a significantly larger number of immunosuppressive network genes was revealed in IDH1 wildtype tumors (such as macrophages, co-inhibition APC, co-inhibition T cells, and pDC markers) when compared with the mutant tumors. Importantly, we found that the key immunoregulatory genes of PD-1 ligands, PD-L1 and PDCD1LG2 (PD-L2) (Keir et al., 2008), were tightly associated with IDH1 mutations in these pLGGs and pGBMs (the PDCD1LG2 results are not shown in this report). Recent clinical applications in cancers have shown that by blocking the interaction of PD-1 and PD-L1, antibodies may unlock activated tumor-reactive T cells and induce a durable antitumor response (Topalian et al., 2012), suggesting that this axis plays a critical role in tumor immunosuppression. We found that the gene expression of PD-L1 was inversely correlated with the OS of patients with pLGGs, but not with that of those with pGBMs. This may be due to the fact that LGGs represent a broader spectrum of PD-L1 gene levels and more polarized survivals than GBMs. Patients with GBMs have a median survival time <15 month, and their tumors are predominantly of the IDH wildtype, in which PD-L1 expression was more uniformly distributed.

We then specifically focused on evaluating the potential consequence of the IDH1 mutation and its impact on the immune landscape in gliomas. We found that a quantity of IDH1-mutant protein expressed in tumors was positively associated with RFS, but not with OS in all tested patients, suggesting that certain factors can transiently control tumor growth as a result of the IDH1 mutation. Previous research has shown that IDH1 mutations result in the production of the oncometabolite 2HG, and the accumulation of this product *in vivo* contributes to the formation and malignant progression of gliomas (Dang et al., 2009). Our study revealed that this product can transiently result in the increased DNA methylation that downregulates the gene and protein expression of PD-L1. Since PD-L1 expression can be influenced by multiple factors, such as interferon (IFN)-γ and interleukin (IL)-4 (Sharpe et al., 2007), more investigations regarding how this molecule is regulated in gliomas are required. Nevertheless, our results suggest that epigenetic alterations in gliomas caused by the IDH1 mutation could be one of the mechanisms of action underlying the distinct immunological features between IDH1-mutant and wildtype tumors.

The impact of IDH mutations in gliomas was found to apparently sensitize the tumors to radio/chemotherapy, as no differences were found in the progression-free survival between IDH-mutant and IDH-WT gliomas if patients were not given radio/chemotherapy from the time of surgery to the time of disease progression (Hartmann et al., 2011; Ahmadi et al., 2012). Therefore, there are treatment options that patients with IDH mutations may consider. Additionally, the recent development of immunotherapy approaches using vaccine or cellular therapy to specifically target the mutation (Mondesir et al., 2016), as well as the potential sensitivity of IDH mutations to PARP inhibitors (Sulkowski et al., 2017), provide additional opportunities through which to treat this group of patients. For wildtype tumors, there are unfortunately no optimal treatment options available. Given the fact that the induction of PD-L1 overexpression is negatively associated with patient OS, and this characteristic has been linked to resistance to anticancer therapies in other human cancers (Wang et al., 2016; Wu et al., 2016; Xu et al., 2016), further understanding of the overexpression of PD-L1 by IDH1 wildtype tumors would provide a useful clinical reference that could be employed to stratify patients to ensure proper use of PD1/PD-L1 blockers in radio/chemo-resistant IDH wildtype gliomas.

## Conclusion

The information gleaned from the methylation status and expression profile of the PD-L1 gene in gliomas is expected to help stratify patients, so that they can receive more appropriate and targeted treatment options, such as immune checkpoint inhibitors, that are specific for IDH1 wildtype gliomas.

## Author contributions

LM, ZL, and JH: conception and design; LM, CY, YL, HG, HT, YC, LJ, and JQ: development of methodology; LM, CY, PK, and JH: data analysis; LM, CY, ES, ZL, and JH: analysis and interpretation of data; LM, YC, GD, AK, ES, DM, ZL, and JH: writing, review, and/or revision of the manuscript; JH: Study supervision.

### Conflict of interest statement

The authors declare that the research was conducted in the absence of any commercial or financial relationships that could be construed as a potential conflict of interest.
