# Supplementary figures and images for: The IDH1 Mutation-Induced Oncometabolite, 2-Hydroxyglutarate, May Affect DNA Methylation and Expression of PD-L1 in Gliomas
Source: Front Mol Neurosci. 2018 Mar 28;11:82. doi: 10.3389/fnmol.2018.00082 (PMC5882817; doi:10.3389/fnmol.2018.00082)

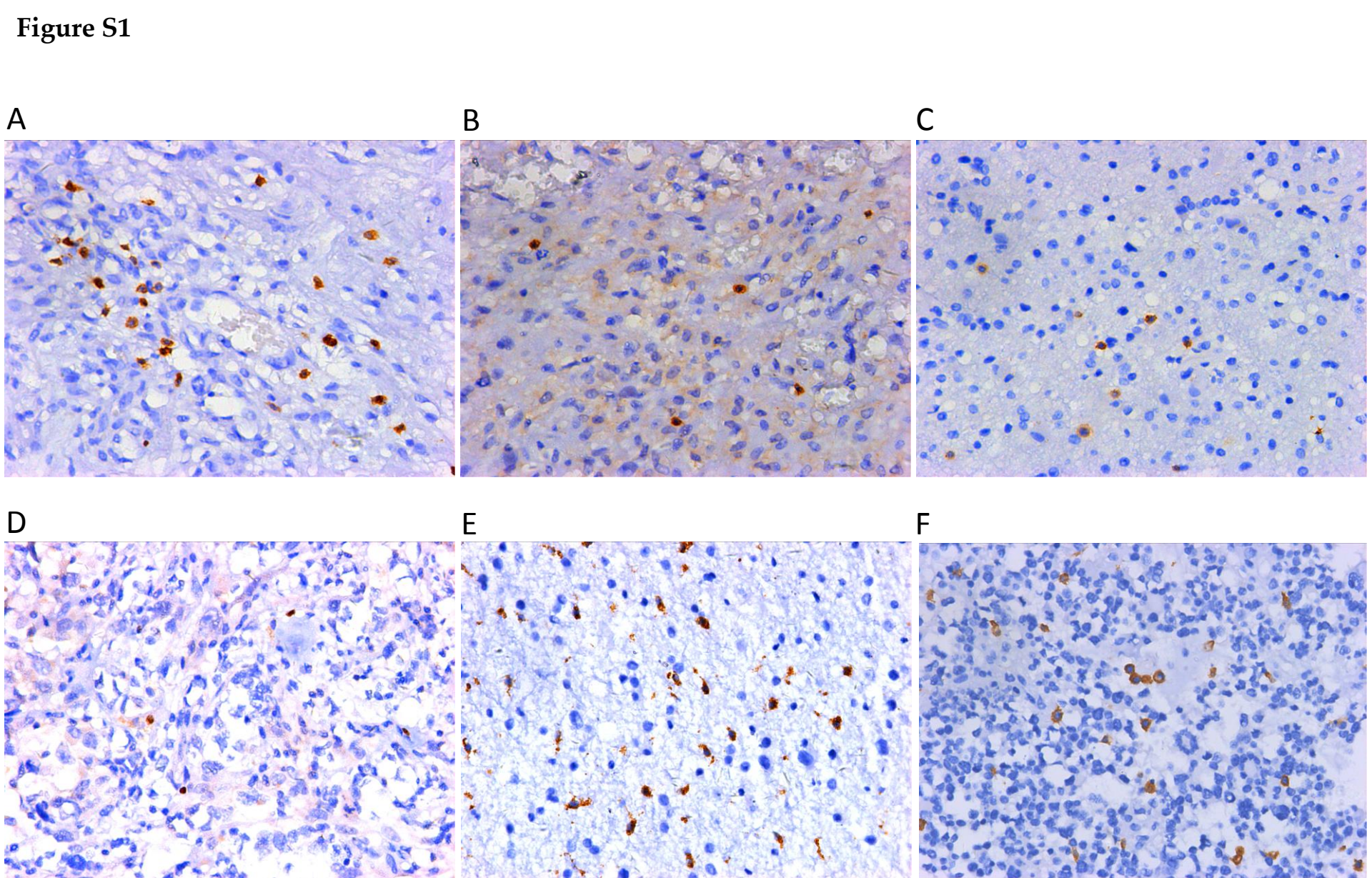

Supplement: Supplementary file 1 [file Image1.PDF]

Figure S2

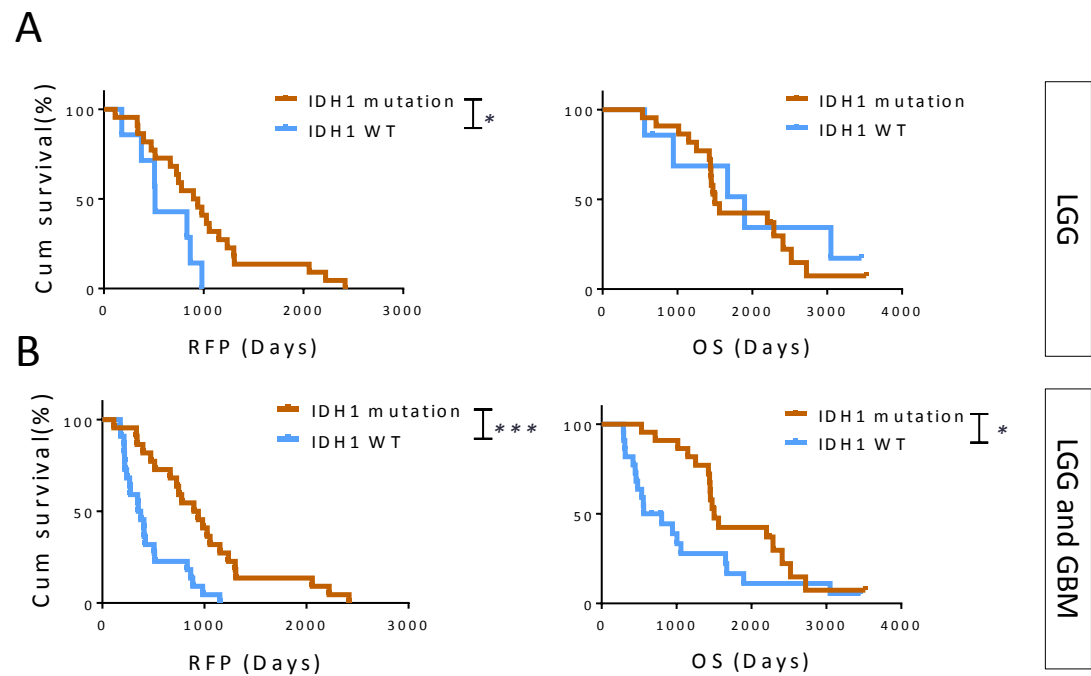

Supplement: Supplementary file 2 [file Image2.PDF]

**Figure S3**

**A**

Primary to Recurrence (LGG-LGG)

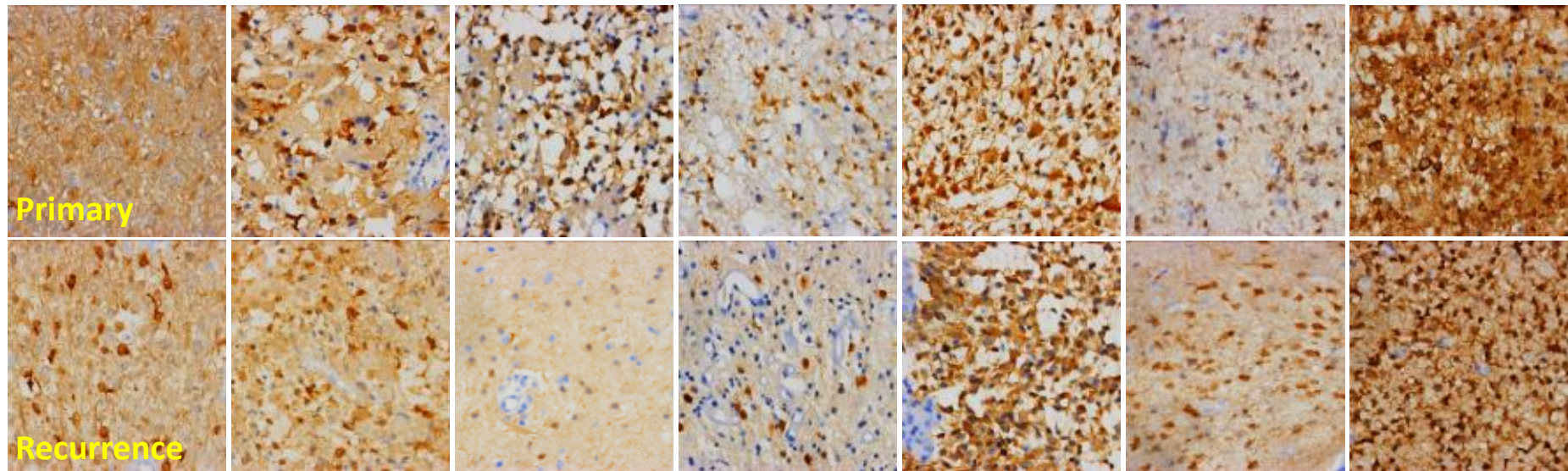

**B**

Primary to Secondary (LGG-GBM)

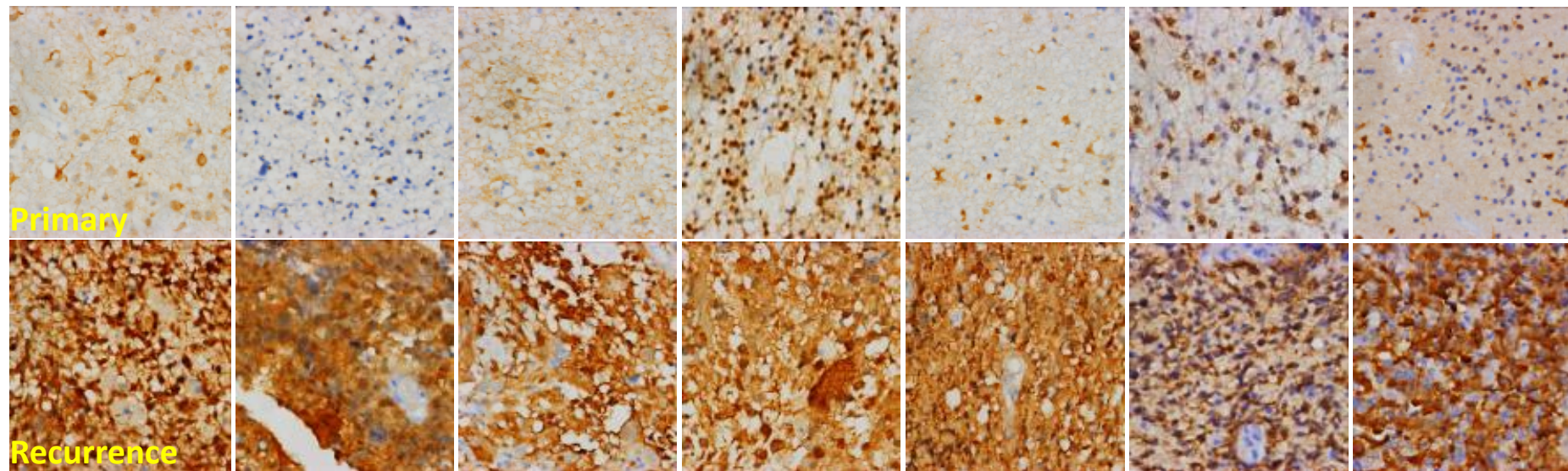

Supplement: Supplementary file 3 [file Image3.PDF]

Figure S4

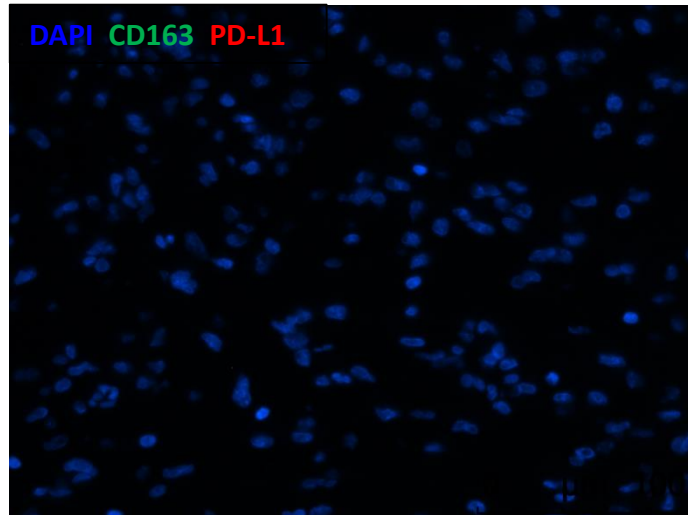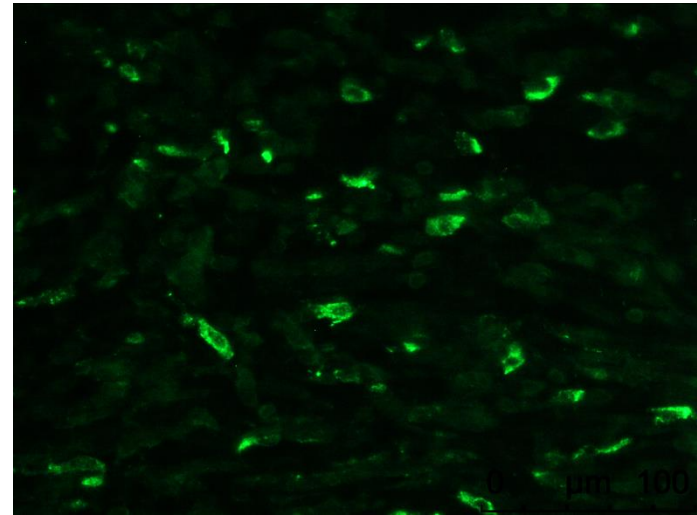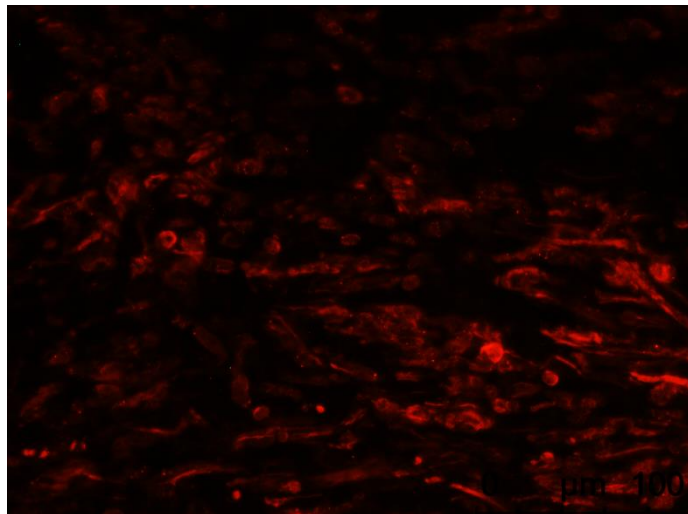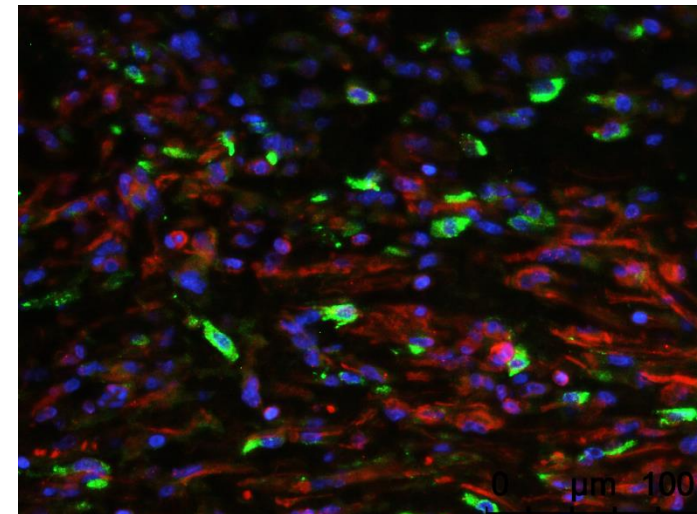

Supplement: Supplementary file 4 [file Image4.PDF]
