# Supplementary material for: The IDH1 Mutation-Induced Oncometabolite, 2-Hydroxyglutarate, May Affect DNA Methylation and Expression of PD-L1 in Gliomas
Source: Front Mol Neurosci. 2018 Mar 28;11:82. doi: 10.3389/fnmol.2018.00082 (PMC5882817; doi:10.3389/fnmol.2018.00082)
Supplement: Supplementary file 5 [file Table1.PDF]

Table S1. Infiltrating immune subsets upon recurrence

Cell subsets	LGG -LGG	LGG-GBM	GBM-GBM
TH1	0.214	0.290	0.102
TH2	0.000	0.000	0.812
CENTER MEMORY CD8 T CELLS	0.210	0.202	0.702
DC	0.208	0.295	0.200
TGD	0.191*	0.211	0.102
MACOPHAGES	0.207	0.479	0.821
EFFECTOR MEMORY CD8 T CELLS	0.470	0.292	0.099
TREG	0.483	0.517	0.600
PDC	0.419	0.406	0.171
IDC	0.503	0.108	0.271
NK	0.711	0.589	0.207
MONOCYTES	0.469	0.108	0.184
ACTIVATED CD8 T CELLS	0.607	0.103	0.398
MAST CELLS	0.630	0.609	0.704
MDSC	0.711	0.292	0.288
T CELLS	0.679	0.907	0.592
ACTIVATED CD4 T CELLS	0.886	0.000	0.821
EOSINOPHIL	0.226	0.088	0.197

* p-values of Spearman test, $p<0.05$ is considered significant between LGG to LGG; LGG to GBM or GBM to GBM recurrence
