# Supplementary material for: The IDH1 Mutation-Induced Oncometabolite, 2-Hydroxyglutarate, May Affect DNA Methylation and Expression of PD-L1 in Gliomas
Source: Front Mol Neurosci. 2018 Mar 28;11:82. doi: 10.3389/fnmol.2018.00082 (PMC5882817; doi:10.3389/fnmol.2018.00082)
Supplement: Supplementary file 6 [file Table2.PDF]

Table S2. Patients Demographic and Clinical Characteristics

Characteristics	LGG → LGG (18-Pair)	LGG → GBM (17-Pair)	GBM → GBM (15-Pair)
**Age at primary surgery (years)**			
Mean	41.7	43.8	45.9
Standard deviation	8.6	11.6	13.4
Range	30-58	29-66	22-68
**Age at recurrent/secondary surgery(years)**			
Mean	43.5	46.6	46.7
Standard deviation	8.5	10.9	13.8
Range	31-59	31-68	23-69
**Gender (Male)**	45(27.8)	5 (29.4)	6(40)
**Race/ethnicity**			
Han	17(94.4)	17 (100)	15(100)
Mongolian	1 (5.6)		
**Pre-primary-surgery treatment**	0	0	0
**Adjuvant therapy after primary-surgery**			
Radiotherapy	11 (61.1)	10 (58.8)	2(13.3)
Chemotherapy (TMZ/other)*	0	0	0
Radiotherapy plus TMZ/other	6(33.3)	7 (41.2)	13(86.7)
**IDH 1 mutation**	14 (77.8)	10 (58.8)	0(0)
**Tumor size in primary surgery**			
Mean	36	32.8	40.2
Standard deviation	11.2	10	11.6
Range	16.66-56.88	12-48	18.56-64.14
**Tumor size in recurrent/secondary surgery**			
Mean	38.3	45.7	50.4
Standard deviation	16.8	17.2	19.9
Range	15-80.3	20.5-85	30-110.01
**Extent of resection in Primary Surgery**			
Gross total resection	18 (100)	16 (94.1)	14(93.3)
Subtotal resection (50-99%)	0	1 (5.9)	1(6.7)
**Recurrence-Free Period (RFP)* (days)**			
Mean	655.9	1046.9	399.7
Standard deviation	372.6	641.3	341
Range	113-1389	319-2417	175-1150
**Overall survival (OS) (days)**			
Mean	1741.7	1382.5	640.3
Standard deviation	960.6	656.3	397.5
Range	531-3050	355-2724	309-1654
**Laterality**			
Left	10 (55.6)	12 (70.6)	11(73.3)
Right	8 (44.4)	5 (41.7)	4(26.7)
**Tumor Location**			
Frontal	7 (38.9)	8 (47.1)	6(40)
Temporal	6 (33.3)	6 (35.3)	2(13.3)
Parietal	2 (11.1)	3 (17.6)	4(26.7)
Occipital	3 (16.7)	0	3(20)
**First presenting symptom**			
Headache	7 (38.9)	7 (41.2)	8(53.3)
Mental status change	1 (5.6)	1 (5.9)	0
Motor or movement change	5(27.8)	3 (17.6)	4(26.7)
Seizure/Epilepsy	3 (16.7)	3 (17.6)	0
Sensory or visual change	2 (11.1)	3 (17.6)	3(20)

* No MGMT methylation information is available for these patients.

** RFP was measured from the date of surgery to the first MRI-confirmed recurrence.
