# Supplementary material for: The IDH1 Mutation-Induced Oncometabolite, 2-Hydroxyglutarate, May Affect DNA Methylation and Expression of PD-L1 in Gliomas
Source: Front Mol Neurosci. 2018 Mar 28;11:82. doi: 10.3389/fnmol.2018.00082 (PMC5882817; doi:10.3389/fnmol.2018.00082)
Supplement: Supplementary file 7 [file Table3.PDF]

Table S3. Primer Sequences used in this study

Gene Name	Gene Bank Accession number	5′ primer	3′ primer
IDH1 （exon 4）	NM_005896	TATTGCCTCTATCT GGTGAAA	AATGGGTGTAGATACCAAAAG
PD-L1(cg15837913)	NM_014143	GGTAGAATATTAGGGATTTTGAGTATTT	CAACAACAAACCCATATAACTTTAAT
PD-L1(cg19724470)	NM_014143	TTGATGTTAGGTTGGAGGTTTG	AAACTCCTCCATTCCTCTTT
PD-L1	NM_014143	TCTGGACAAGCAGTGACCATC	GTGTTGATTCTCAGTGTGCTGG
