# Supplementary material for: The IDH1 Mutation-Induced Oncometabolite, 2-Hydroxyglutarate, May Affect DNA Methylation and Expression of PD-L1 in Gliomas
Source: Front Mol Neurosci. 2018 Mar 28;11:82. doi: 10.3389/fnmol.2018.00082 (PMC5882817; doi:10.3389/fnmol.2018.00082)
Supplement: Supplementary file 8 [file Presentation1.pdf]

The IDH1 Mutation-Induced Oncometabolite, 2-Hydroxyglutarate, May Affect DNA Methylation and Expression of PD-L1 in Gliomas

Luyan Mu [1,2], Yu Long [1,3], Changlin Yang [3], Linchun Jin [1,3], Haipeng Tao [1,3], Haitao Ge[1], Yifan Chang [3]†, Aida Karachi [3], Paul S. Kubilis [3], Gabriel De Leon[3]†, Jiping Qi [4], Elias J. Sayour [3], Duane A. Mitchell [3], Zhiguo Lin[1]* and Jianping Huang[3]*

[1]The Fourth Section of Department of Neurosurgery, The First Affiliated Hospital, Harbin Medical University, Harbin, China, [2]The First Section of Department of Neurosurgery, The Fourth Affiliated Hospital, Harbin Medical University, Harbin, China, [3]Lillian S. Wells Department of Neurosurgery, University of Florida, Gainesville, FL, United States, [4]Department of Pathology, The First Affiliated Hospital, Harbin Medical University, Harbin, China

† Present address: Yale School of Medicine, New Haven, Connecticut, USA.
Department of Molecular and Structural Biochemistry, North Carolina State University, Raleigh, North Carolina, USA.

Running Title: PD-L1 expression in gliomas

✉ **Correspondence**:
Dr. Zhiguo Lin (linzhiguo@hotmail.com). Address 23 Youzheng St. Harbin, China, 150001. Dr. Jianping Huang (jianping.huang@neurosurgery.ufl.edu). Address: P.O. Box 100265, 1149 Newell Dr. Gainesville, FL 32610. Tel: +1-352-273-6835, Fax: +1-352-392-8413

Supplementary Figure Legends

Figure S1 Representative images of immunohistochemistry staining for CD3+ (A), CD4+ (B), CD8+ (C) and Foxp3+ (D)T cells, as well as CD68+ (E) and CD163+ (F) macrophages in primary GBM samples. The images were taken at 20x magnification.

Figure S2 Association of IDH1 mutation status in primary tumors and patients (see **Table S2**) clinical outcomes. (A) Patients in LGG group were stratified based on their tumor IDH1 mutation status, and Kaplan Meier survival analysis was performed for RFP and OS. (B) Same analysis for patients, including LGGs and GBMs. The log-rank test was used to compare the groups.

Figure S3 Representative images of mutant IDH1 protein in primary and recurrent gliomas. (A-B) Paired (7 per group) primary and recurrent gliomas from LGG-LGG and LGG-GBM groups were stained by immunohistochemistry using IDH1 mutant protein specific antibody. 20x magnifications were used for the images.

Figure S4 No PD-L1 expression on tumor-infiltrating CD163+ macrophages in gliomas. Images of PD-L1 and CD163 in an LGG. The data were reprehensive of one of three tests.

Table S1 Infiltrating immune subsets upon recurrence.

Table S2 Patients Demographic and Clinical Characteristics

Table S3 Primer Sequences used in this study
